# Boosting scientific community values: The impact of social inclusion interventions on biomedical instructors

**DOI:** 10.1017/cts.2025.81

**Published:** 2025-04-28

**Authors:** Paul R. Hernandez, Wenyi Du, Cristian Cervantes Aldana, Nichole A. Broderick, Jo Handelsman, Hyewon Lee, Linlin Luo, Natalia Maldonado, Holly A. Miller, Sarah Miller, Megan Patterson, Rachelle Pedersen, Perla Sandoval, Janice Vong, Mica Estrada

**Affiliations:** 1 Department of Teaching, Learning, and Culture, Texas A&M University, College Station, TX, USA; 2 Department of Social and Behavioral Sciences, University of California San Francisco, San Francisco, CA, USA; 3 Department of Biology, Johns Hopkins University, Baltimore, MD, USA; 4 Wisconsin Institute for Discovery, University of Wisconsin–Madison, Madison, WI, USA; 5 Department of Plant Pathology, University of Wisconsin–Madison, Madison, WI, USA; 6 Department of Health Behavior, Texas A&M University, College Station, TX, USA; 7 Department of Curriculum & Instruction, Texas Tech University, Lubbock, TX, USA

**Keywords:** Mentoring, faculty, higher education, STEMM, TIMSI

## Abstract

Interventions to foster inclusive learning environments may benefit college STEMM instructors (NASEM, 2019). We investigated the impact of a social inclusion intervention (SII) on scientific self-efficacy, identity, community values, and persistence intentions in a large and diverse sample of biomedical college instructors (*n* = 116) in the USA. The results indicated that the SII group developed stronger scientific community values than the control group, and the effect was the strongest for instructors who had initially expressed lower values. From a mentoring perspective, the intervention helps boost feelings of community values, which is linked to increased persistence in STEMM careers.

## Introduction

Positive professional relationships are critical for building community and belonging in academia, especially for faculty. Mentorship plays a vital role in education, with funding agencies calling for high-quality mentorship in higher education [1]. Experienced mentors provide faculty mentees with valuable guidance gained through their own experience [2]. National trends highlight racial/ethnic disparities at all academic career levels in science, technology, engineering, mathematics, and medicine (STEMM) fields [3]. Mentoring may be an effective intervention to support the persistence of persons from underrepresented groups (e.g., African Americans, Hispanics, American Indians/Alaska Natives, and Native Hawaiians/Pacific Islanders) in biomedical fields [4], at undergraduate [5] and faculty [6] levels. Although mentorship’s benefits on faculty are well documented, limited research explores *how* mentorship affects their social integration into their disciplinary communities.

### Theoretical framework

The Tripartite Integration Model of Social Influence (TIMSI) explains why and how individuals integrate into their scientific communities through three orientations [7]: scientific self-efficacy, identity, and internalization of community values. Scientific self-efficacy reflects confidence in performing science-related tasks; identity involves belonging to and identifying with the scientific community; and community values concern accepting and aligning with the values of the external scientific community with one’s personal values. Studies indicate these orientations were correlated with career choices in STEMM [8,9], with a longitudinal study showing that science identity predicted persistence even four years after undergraduate graduation [8].

Faculty mentors can reinforce scientific community values, strengthening ties for both student and faculty mentees. Research with graduate students and early-career faculty indicates that internalizing these values uniquely predicts STEMM career persistence [10], emphasizing the importance of value orientation when making longer-term career choices. While faculty generally achieve a high level of professional/disciplinary integration, social integration remains an ongoing process that requires continuous effort. National research found systematic patterns of faculty departures, particularly among those from underrepresented groups [11], highlighting the need to understand better factors that support sustained commitment to disciplinary communities. Faculty integration levels can vary, and increased scientific self-efficacy, identity, or community values would signal an enhanced connection to the scientific community. Since that perceived value misalignment is linked to faculty attrition, endorsing scientific community value may be especially important for long-term retention and engagement in academic STEMM communities.

### Peer mentorship in higher education

In mentorship theory, a “mentee” refers to a less experienced individual, and “mentor” describes a more experienced individual [2]. Mentorship is a cooperative process whereby mentors support mentees’ professional growth through psychosocial and career support functions [1]. Mentorship is critical for professional development and retention in higher education, with peer-mentoring programs shown to boost motivation for research and an inclusive, collaborative culture [1,6]. Prior research indicates mentoring has numerous positive outcomes, such as increased self-efficacy [12] and network size [13].

Mentorship is most effective when mentees engage with a network of supporters rather than a single mentor [1]. Expanded networks provide diverse insights on career advancement, well-being, and academic achievement [13]. Building on prior work, this study examines how a brief intervention affects TIMSI factors and STEMM persistence intentions among faculty/instructors (hereafter referred to as instructors) in a Biology curriculum training program (Tiny Earth). This program facilitated peer mentorship and expanded instructors’ support networks, which was an ideal context to assess the impact of the intervention on social integration.

### Improving mentorship through similarity interventions

Several studies have explored ways to improve mentorship quality through perceived similarity, a stronger predictor of high-quality mentorship than demographic similarity [2]. Perceived similarity, also referred to as psychological or deep-level similarity, indicates the extent to which a mentee feels they share common attributes, values, beliefs, or personality with their mentor [2]. A key example is the “Creating Birds of a Feather” approach, which experimentally tested the effect of displaying shared life experiences, preferences, hobbies, and interests between instructors and students (e.g., both like watching movies in their free time) to promote relationship quality [14,15]. Research indicates that those who were made aware of shared similarities experienced more positive relationship and mentorship outcomes [15]. In recent college classroom studies, this intervention boosted students’ perceptions of similarity with their instructor [14,15], and one study found that this was particularly important for students who initially held lower similarity beliefs [15].

### Current study

While mentorship in higher education has substantial benefits, there remains a need for rigorous testing of how peer mentorship influences instructor mentees’ social integration into STEMM [6]. This study addresses this gap using a randomized longitudinal pretest (T1, T2, T3) - posttest (T4) design to test the effectiveness of a Social Inclusion Intervention (“SII,” described in the Method section). We hypothesized that mentees receiving the SII would report greater integration, evidenced by increased science-related self-efficacy, identity, community values, and intentions to persist in their STEMM careers. Since the sample consisted of biomedical science faculty already committed to STEMM, persistence intentions are a proxy for career satisfaction and/or turnover intentions. This study examined the following research questions (RQ):

1. Does the SII increase posttest science-related self-efficacy, identity, community values (i.e., TIMSI factors), and intention to persist in their STEMM careers, controlling for pretest values?
2. Are the impacts of the SII on posttest TIMSI factors moderated by pretest scores?

## Materials and methods

### Participants

Biomedical science college instructors in a course-based undergraduate research experience (CURE) curriculum training program participated in an NIH-funded national longitudinal study. Of the 146 instructors, 29 were excluded for missing information. Analysis confirmed data Missing Completely at Random (*χ* ^2^(4) = 4.01, *p* = .40). The analytic sample included 116 instructors (58 control, 58 SII), 76% identified as women, and 18% from underrepresented racial/ethnic groups (Table 1).

**Table 1.** Instructors’ demographics as a function of treatment condition (*N* = 116, *n* = 58 per group)

	Treatment (SII)	Control
	*n (%) or M (SD)*	*n (%) or M (SD)*
**Gender**		
Woman	43 (37.07)	45 (38.79)
Man	14 (12.07)	12 (10.34)
Transgender	0 (0.00)	0 (0.00)
Non-binary	0 (0.00)	1 (0.86)
Other	0 (0.00)	0 (0.00)
**Race/Ethnicity**		
White, Non-Hispanic	47 (40.52)	46 (39.66)
Hispanic or Latinx	5 (4.31)	4 (3.45)
Asian	3 (0.35)	1 (0.86)
Black/African American	0 (0.00)	5 (4.31)
Middle Eastern or North African	1 (0.86)	0 (0.00)
Native Hawaiian or Pacific Islander	0 (0.00)	0 (0.00)
American Indian or Alaska Native	0 (0.00)	0 (0.00)
Multiracial	1 (0.86)	1 (0.86)
Other	1 (0.86)	1 (0.86)
	

*Note*: Instructors self-reported their demographic data as part of the survey administered at pretest. SII = social inclusion intervention; Latinx = Latino/a and/or non-binary Latin American/Spanish origin. One participant in the SII group chose not to report their gender.

### Procedure

This randomized intervention study took place in the context of the training program to support college life science instructors’ incorporation of a CURE curriculum into their courses (see supplemental materials for a full description). Instructors apply to participate in a one-week training program, and the training program staff determines admission. Instructors accepted to participate in the program attended a week-long training to incorporate CURE into their curricula (note: a shift to online training occurred during COVID-19). Only after being accepted into the training program were attendees recruited to participate in the present study.

Instructors who consented to participate were randomly assigned to standard or standard training plus the SII. The SII consisted of two brief psychological strategies: Academic and Professional Ecosystem Mapping Activity [13] and Creating Birds of a Feather Activity [14]. Briefly, the mapping activity, which was adapted from a prior study of mentoring undergraduate women in male-dominated STEM fields [16], aimed to broaden instructors’ perspectives on mentorship and strategies to grow their mentor network. The CBoF activity, informed by research [14], was designed to enhance mentor-mentee similarity to establish authentic social connection and experience of inclusion (complete details for the interventions are included in the Supplemental Materials). The study used an “intent-to-treat” design and did not collect information on intervention adherence. Both groups completed pre- and post-test surveys via Qualtrics. Participation at each timepoint was incentivized with a $20 Amazon or Starbucks gift card. The University of California, San Francisco Institutional Review Board approved the study procedures (IRB #19-28867).

### Measures and data collection

Data were collected via Qualtrics surveys using a Tailored Panel Management approach [17]. The scores for persistence intentions [18], and the TIMSI measures [7] of scientific self-efficacy, identity, and community values were created by averaging composite scores across T1, T2, and T3 to capture participants’ average pretest status (complete information is in the Supplemental Materials). For example, pretest scientific self-efficacy was the average of T1, T2, and T3 scores. We took the average of the three pretest measures because they were administered over a short time and before the intervention; therefore, we did not expect a change in their beliefs. Our approach to aggregating the three pretests into a single pretest assessment also allowed us to avoid losing a small number of cases with missing data at one of the pretest time points (*n* = 13). All outcomes were assessed six months post-training (T4, posttest), with higher mean scores indicating greater levels of each construct. Descriptive statistics and reliability coefficients appear in Supplemental Table 1.

## Results

**Figure 1.**
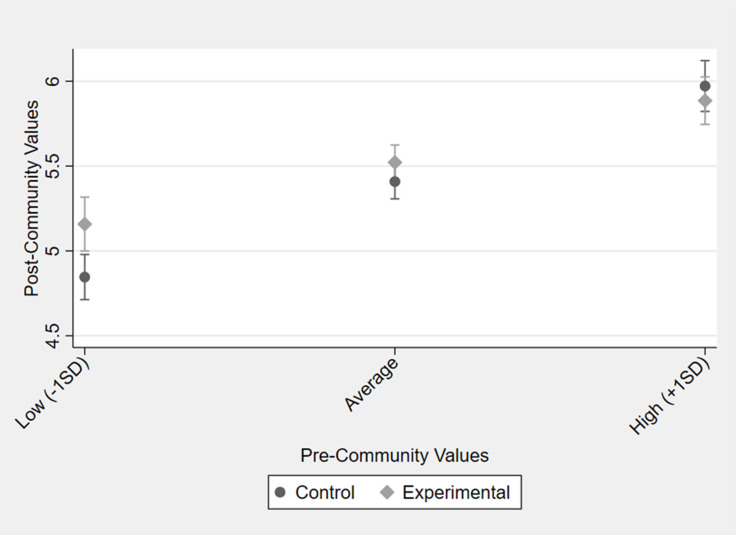
Effect of treatment status on posttest scientific community values moderated by pretest scientific community values. SII = social inclusion intervention.

Preliminary independent *t*-tests showed that the control and SII intervention groups did not differ on the TIMSI variables at baseline (see Supplemental Table 1 notes). Next, multiple regression analyses in Stata v18 indicated that the SII group exhibited significantly higher posttest scientific community values than the control group (RQ 1; Table 2). However, no other group differences emerged for scientific self-efficacy, identity, or persistence intention. Finally, moderated regression analyses showed that pretest scientific community values moderated the SII status—posttest scientific community values relationship (RQ2; Table 2). A simple slope graph revealed that among instructors who expressed lower scientific community values at the pretest, the SII group reported higher average community values at the post-test than the control group (Figure 1). There were no group differences in posttest scientific community values among instructors who expressed higher values at the pretest.

**Table 2.** Summary of the moderated regression models predicting TIMSI factors and persistence intentions (*N* = 116)

	Scientific identity^c^			Scientific self-efficacy^d^			Scientific community values^e^			Persistence intentions^f^		
Predictors	*b*	*SE*	*95% CI*	*b*	*SE*	*95% CI*	*b*	*SE*	*95% CI*	*b*	*SE*	*95% CI*
Intercept	**1.13** ^*^	**0.45**	**[0.25, 2.01]**	0.40	0.58	[−0.75, 1.54]	−0.31	0.51	[−1.32, .69]	0.04	1.12	[−2.18, 2.26]
SII^a^	−0.14	0.59	[−1.32, 1.03]	0.88	0.74	[−0.58, 2.33]	**1.61** ^**^	**0.71**	**[0.19, 3.02]**	−0.57	1.41	[−3.37, 2.22]
Pretest^b^	**0.74** ^***^	**0.11**	**[0.53, 0.95]**	**0.91** ^***^	**0.14**	**[0.63, 1.18]**	**1.04** ^***^	**0.09**	**[0.86, 1.23]**	**0.95** ^***^	**0.13**	**[0.69, 0.12]**
SII × Pretest^b^	0.04	0.14	[−0.24, 0.31]	−0.22	0.18	[−0.57, 0.13]	**−0.27** ^**^	**0.13**	**[−0.53, −0.02]**	0.07	0.16	[−0.26, 0.39]

*Note*: SII = social inclusion intervention; 95% CI = 95% confidence interval. Results in **bold** indicate that it was statistically significantly different from zero.

a Reference group is Control.

b Status_pre indicates the initial status for each variable (i.e., scientific identity at the pretest, scientific self-efficacy at the pretest, scientific community values at the pretest, or persistence intentions at the pretest).

c *F*(3, 112) = 44.85, *p* < 0.001, *R* ^*2*^ = .55.

d *F*(3, 112) = 28.46, *p* < 0.001, *R* ^*2*^ = .43.

e *F*(3, 112) = 60.66, *p* < 0.001, *R* ^*2*^ = .62.

f *F*(3, 112) = 51.64, *p* < 0.001, *R* ^*2*^ = .58.

* *p* < 0.05, **p < 0.01, ***p < .001.

## Discussion

Effective mentorship is critical for maintaining interest in STEMM [1]. This study developed a Social Inclusion Intervention to promote TIMSI measures of integration and persistence in STEMM among biomedical instructors through peer mentorship. It extended prior research on social inclusion by examining its effects on faculty. While this is the first study to apply such an intervention using TIMSI, several limitations should be noted. Career stage (e.g., early) and positions (e.g., teaching faculty) may have influenced responses, but this data was not collected. TIMSI and persistence intention measures, designed for students, may require adaptation for faculty, particularly regarding self-efficacy. Additionally, adherence to the intervention was not tracked, leaving the extent of mentoring discussions of similarity unknown. Institutional differences in resources, time, and buy-in were also not examined. This study took place in part during the COVID-19 pandemic and when there were many challenges regarding race relations and political unrest. Persistence intentions decreased in both the SII and control groups, which may indicate that faculty enthusiasm for their profession might have been negatively impacted. Despite these limitations, the study provides novel insights into faculty mentoring interventions and the role of social inclusion on STEMM persistence.

Our findings indicate that the SII mapping and similarities activities increased endorsement of scientific community values, a key social integration predictor of persistence [7,8]. This aligns with prior research showing that mentors share cultural values with mentees and positively influence their scientific community values [10]. Notable, the SII had the strongest longitudinal effect on those with initially low scientific community values. Since research interest decreases among early-career STEMM professionals [19], SII may help boost instructors’ social integration and commitment, particularly for those with weaker internalized values. Although our study did not examine why participants had lower initial values, past research indicates gender and ethnicity differences in academic achievement value development [20], which may be mirrored in TIMSI values. TIMSI emphasizes that academic scientific mentors play an important role in fostering scientific community values in mentees. Connecting instructors with mentors who embrace scientific community values may support instructors’ development and internalization of community values. Future research should examine the long-term benefits of sustaining higher scientific community values on career satisfaction and advancement.

The SII did not impact scientific self-efficacy, identity, or persistence intentions, unlike findings in undergraduate STEM populations [8,9]. This difference likely reflects developmental differences between undergraduate and PhD-level instructors. Our findings indicate that while instructors had high scientific efficacy and identity, their endorsement of scientific values varied before the intervention. Prior research indicates value endorsement is a stronger predictor of integration than efficacy and identity in graduate and early-career faculty [10]. This may explain why the SII had a greater impact on value endorsement at this career stage. Alternatively, variability in peer-mentoring quality—a key factor linking mentoring interactions to TIMSI measures and persistence [2]—may have obscured the SII’s effects. Future research should investigate mentoring quality as a potential mediator linking the SII and key outcomes.

In summary, this work highlights the potential of SII-type interventions to support biology instructors’ continued integration and persistence. Such interventions may be particularly important for retaining faculty who experience individual or cultural challenges to values alignment in STEMM.

## Supporting information

Hernandez et al. supplementary material Hernandez et al. supplementary material

## Data Availability

The data that support the findings of this study are openly available in the Texas Data Repository at https://doi.org/10.18738/T8/DV1GGB

## Supplementary material

The supplementary material for this article can be found at https://doi.org/10.1017/cts.2025.81.
