# Supplementary material for: Boosting scientific community values: The impact of social inclusion interventions on biomedical instructors
Source: J Clin Transl Sci. 2025 Apr 28;9(1):e105. doi: 10.1017/cts.2025.81 (PMC12171922; doi:10.1017/cts.2025.81)
Supplement: Hernandez et al. supplementary material [file S2059866125000810sup001.pdf]

Supplemental Materials for:

Boosting Scientific Community Values: The Impact of Social Inclusion Interventions on Biomedical Instructors

Author Information

[1*]Paul R. Hernandez (https://orcid.org/0000-0002-4063-357X)
[1]Wenyi Du (https://orcid.org/0000-0003-4737-9268)
[2]Cristian Cervantes Aldana (https://orcid.org/0000-0001-7689-8062)
[3]Nichole A. Broderick (https://orcid.org/0000-0002-6830-9456)
[45]Jo Handelsman (https://orcid.org/0000-0003-3488-5030)
[1]Hyewon Lee (https://orcid.org/0000-0003-0710-9108)
[1]Linlin Luo (https://orcid.org/0000-0002-3648-4727)
[2]Natalia Maldonado, (https://orcid.org/0009-0009-7522-3966)
[1]Holly A. Miller (https://orcid.org/0000-0002-2344-8989)
[4]Sarah Miller (https://orcid.org/0000-0003-3348-4470)
[6]Patterson, Megan (https://orcid.org/0000-0001-6876-2931)
[17]Rachelle Pedersen (https://orcid.org/0000-0001-8530-9071)
[2]Perla Sandoval (https://orcid.org/0009-0009-9281-2084)
[2]Janice Vong (https://orcid.org/0000-0002-3116-9605)
[2]Mica Estrada (https://orcid.org/0000-0003-3933-2929)

Affiliations

[1]Department of Teaching, Learning, and Culture, Texas A&M University, College Station, TX 77843
[2]Department of Social and Behavioral Sciences, University of California San Francisco, San Francisco, CA 94118
[3]Department of Biology, Johns Hopkins University, Baltimore, MD 21218
[4]Wisconsin Institute for Discovery, University of Wisconsin–Madison, Madison, WI 53715
[5]Department of Plant Pathology, University of Wisconsin–Madison, Madison, WI 53715
[6]Department of Health Behavior, Texas A&M University, College Station, TX 77843
[7]Department of Curriculum & Instruction, Texas Tech University, Lubbock, TX 79409

*Correspondence should be addressed to Dr. Paul R. Hernandez, prhernandez@tamu.edu

This document includes:

Contents

Materials and Methods 3

Procedure 3

Recruitment and Assignment 3

Social Inclusion Intervention Content 4

Part 1: Academic and Professional Ecosystem Mapping Activity (Hernandez et al., 2020) 4

Part 2: Creating Birds of a Feather (Robinson et al., 2019) 10

Creating Birds of a Feather Questionnaire 11

Complete Instruments of Outcome Measures 15

Intentions to Persist (adopted from Woodcock et al., 2016) 15

Science Self-Efficacy (adopted from Estrada et al., 2011) 15

Science Identity (adopted from Estrada et al., 2011) 15

Scientific Community Values (adopted from Estrada et al., 2011) 16

Supplemental Table 1 17

Data availability statement: 18

Materials and Methods

Procedure

Recruitment and Assignment

Recruitment of training program attendees into the current study occurred each semester, from Fall 2019 to Spring 2022, for a total of six cohorts. All attendees accepted into the CURE training program were invited to participate in the study. A month before training (Time 1 or T1), at the time of recruitment, all applicants received an online Qualtrics survey and voluntarily consented to participate in the study. The T1 survey included demographics, the CBoF "getting to know you" questionnaire, and TIMSI and STEMM persistence intentions measures. A week before the training (T2), participants were surveyed again on TIMSI and persistence intentions measures. After the week-long training on CURE techniques and pedagogy, participants completed a third survey (T3) with TIMSI and STEMM persistence intentions measures. In the T3 survey, the SII group completed the mapping activity and was introduced to a veteran CURE peer mentor, along with up to five similarities from the CBoF questionnaire. Participants in the standard training condition were introduced to a veteran CURE peer mentor without CBoF similarities.

Post-training (i.e., between T3 and T4), all instructors were paired with a veteran CURE mentor and introduced via email. For the SII group, these introductions highlighted shared life experiences, preferences, and hobbies from the CBoF "getting to know you" questionnaire.

Six months post-training (T4), participants completed a follow-up survey containing TIMSI and persistence intention measures.

Social Inclusion Intervention Content

Part 1: Academic and Professional Ecosystem Mapping Activity (Hernandez et al., 2020)

The mapping activity, which was adapted from a prior study of mentoring undergraduate women in male-dominated STEM fields [16], aimed to broaden instructors' perspectives on mentorship by utilizing the following components [13]: (a) redefining mentorship to include a network of supporters, (b) reflecting on their current support types, (c) identifying people who provide these supports, (d) creating a mentor map that outlines support types and gaps, and (e) creating an action plan to address gaps and strengthen network diversity and connectivity. In the T3 survey, SII group members completed this mapping activity via multiple-choice questions that built a personalized network map across three main categories: academic support (e.g., intellectual community, professional development), career advancement support (e.g., role models, networking), and personal/emotional support (e.g., values, kindness, and belonging; see Supplemental Material for the complete details).

The following section introduces the activity to participants.

Research indicates that professionals with a diverse network of people who provide them support are more likely to be successful in their career pursuits, in comparison to relying on the myth that we only need a single "super mentor" to provide all the support we need. The following section describes the supports that may be helpful to successful academic careers. Please read each and rate how important you think these different types of support are to being successful in your profession.

The following section asks participants to rate different types of support on a 5-point scale ranging from 1 = not important to 5 = extremely important.

These types of **support help people more successfully get their job done**. In your opinion, how important is each type of support to success in academia?

- Intellectual community support includes getting advice on classes to take, helping you study for tests, working together to complete a group project, or providing feedback on an application to an internship.
- Professional development support includes things that aren't part of your coursework but help prepare you for your future career, such as time-management skills, conflict resolution skills, public speaking training, or training to gain new scientific skills outside of the classroom.

These types of **support that help advance people's academic and professional careers**. How important are these each to you now?

- Networking & sponsorship support can come in the form of people that make sure you know about upcoming workshop, scholarship, or internships, people that will introduce you to influential people, or write you a letter of recommendation.
- Role models are individuals that you want to emulate in one or more aspects of your life, such as school-life balance, productivity, career choices, or influence in the field.

These types of **support help personal and emotional well-being**. How important are these each to you now?

- Safe space support includes people who encourage you to speak your truth, vent frustration without judgment, and help you reframe and compose yourself in order to deal with problems in a professional manner.
- Kindness support are people who convey friendliness and other gestures that affirm that you belong in your field of study.

- Values support includes people who affirm your values and may share your cultural, spiritual or regional experiences)

The following section asks participants to watch a short video.

Please turn on your sound to watch this 90-second video that will provide instructions on the next set of questions and possibly enrich your Tiny Earth experience (If the video doesn't start automatically, hit the play button to begin).

https://youtu.be/nQ8yI9i5pvw

The following section asks participants to create a professional ecosystem map of their mentors.

Now it's your turn to assess your professional ecosystem. Take a moment to think about how support might make a huge difference for your career and well-being. Please fill in the first and last initials of the people that provide you with support to achieve your academic and professional goals. You may list as many people as you like (although most individuals list fewer than ten people that provide them support).

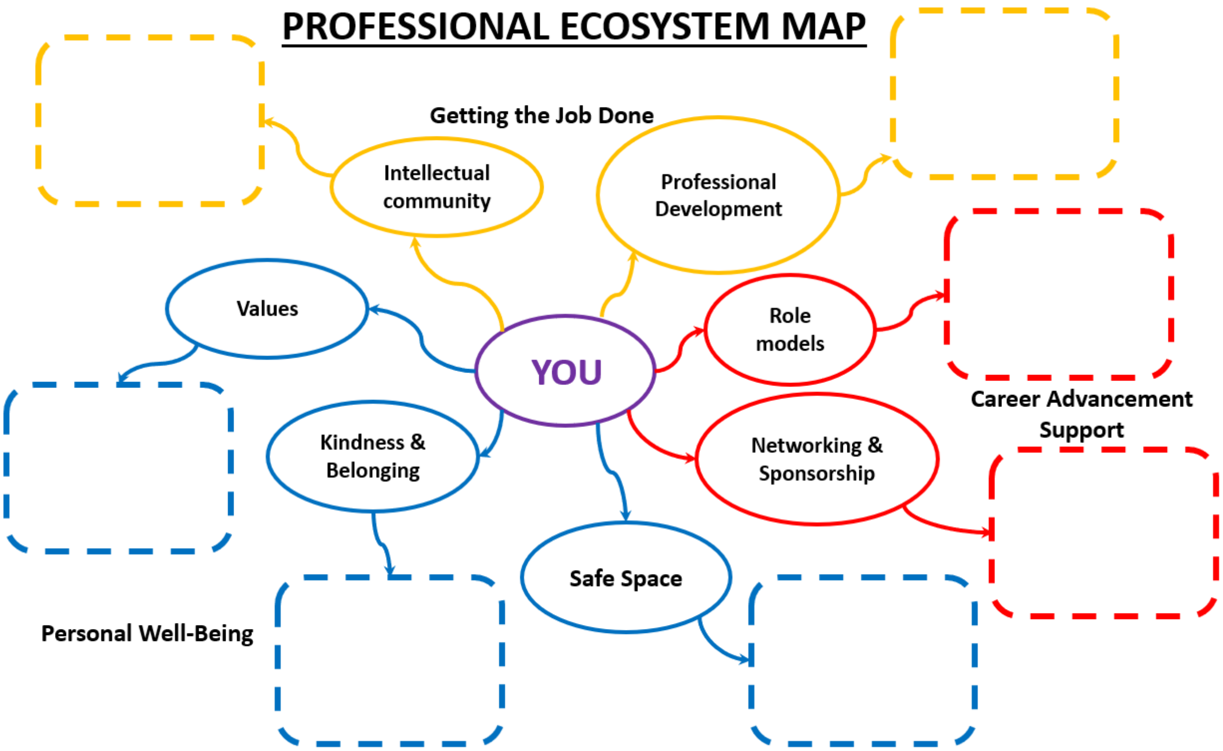

The following sections asks participants to reflect upon the below questions. No responses are collected.

Take a moment to think about your own professional ecosystem map (see bottom of the page), and consider the following questions:

- What kinds of supports do you need?
- What kinds of supports do you think are most important to you personally and professionally?
- Are there any categories that are not important to your career right now?
- Do you feel as if you are getting the support you need?
- In which areas do you have the most people who are willing to support you?

The following section asks participants to reflect upon what support they would like to increase in the future.

Reflecting on your map now, please click the boxes of the categories for which you would you like to add support to your professional ecosystem.

- Intellectual Community
- Professional Development
- Role Models
- Networking & Sponsorship
- Safe Space
- Kindness & Belonging
- Values

Please click the boxes of the categories for which you feel satisfied with the support to your professional ecosystem.

- Intellectual Community
- Professional Development
- Role Models
- Networking & Sponsorship
- Safe Space
- Kindness & Belonging
- Values

Research shows that having a concrete plan, helps us make things happen. Consider potential strategies to grow your academic & professional ecosystem. Can you make some specific, measurable, attainable, realistic, and time-bound goals? Please list 3 strategies you have for strengthening your professional ecosystem.

Strategy 1: ____

Strategy 2: ____

Strategy 3: ____

When will you implement these strategies?

Part 2: Creating Birds of a Feather (Robinson et al., 2019)

Research highlights the role of psychological similarities (e.g., shared attitudes and beliefs) in promoting high-quality mentoring relationships [2]. The CBoF activity, informed by research [14], was designed to enhance mentor-mentee similarity by collecting data on life experiences, preferences, hobbies, or interests that are known to impact quality relationship development. This intervention aims to increase perceptions of similarity to facilitate the establishment of authentic social connection and experience of inclusion. The CBoF activity involves two steps. First, a "getting to know you" questionnaire, was administered to mentors and mentees. The questionnaire consists of 21 questions based on four general commonalities categories: interests, personal, values, and academics. For example, an item in the interest category asks participants: "For your ideal trip, you would: (a) go to a museum, (b) go to a sports event, (c) go to a music concert, (d) go hiking;" And an item in the value category asks, "What do you think is the most important quality in a significant other? (a) kindness, (b) intelligence, (c) honesty, (d) sense of humor." Second, after completing the questionnaire, the research team would prepare an email introduction to each mentor-mentee dyad highlighting up to five similarities from the questionnaire.

In the present study, mentors and mentees completed identical CBoF "getting to know you" questionnaires at T1. After the training (T3), the research team sent each matched mentor-mentee pair in the SII group an email introduction highlighting up to five similarities from the questionnaire.

Mentors and mentees fill out the same list of Creating Birds of a Feather questions (21 items in total). The CBoF questionnaire contains four categories of questions: interests (Q1-Q5), personal (Q6-Q9), values (Q10-14), and academics (Q15-Q21).

Creating Birds of a Feather Questionnaire

The following questions will ask you about your interests, hobbies, and preferences to get to know you and your Tiny Earth Partner Instructors better.

1. If you could go to one sporting event, which of the following would you go to?
   A. Soccer World Cup
   B. Olympics
   C. NBA Championship
   D. Super Bowl
   E. World Series
   F. I'd give my tickets to someone who cared about sports

2. On a day off school and/or work, which of the activities are you most likely to do?
   A. Play sports
   B. Hang out with friends
   C. Watch TV/Movie
   D. Read a book

3. Which of the following activities do you most like to do in your free time?
   A. Exercise
   B. Play games (e.g., board games or video games)
   C. Shopping
   D. Use social media

4. I volunteer in the community.
   A. No
   B. Yes

5. For your ideal trip, would you…
   A. Go to a museum
   B. Go to a sports event
   C. Go to a music concert
   D. Go hiking

6. Do you have a family member or close friend who is in the military?
   A. No
   B. Yes - family member
   C. Yes – friend

7. Which of the following languages do you speak (even just a little bit)?
   A. Arabic
   B. Chinese
   C. French
   D. German
   E. Japanese
   F. Korean
   G. Spanish
   H. Other (text entry)

8. My sense of humor is best described as...
   A. Witty
   B. Goofy
   C. I'm pretty serious

9. Which of the following traits do you think best describe you?
   A. Funny
   B. Caring
   C. Knowledgeable
   D. Outgoing
   E. Hardworking

10. The most important quality in a friend is…
    A. Being there when you need them
    B. Listens to you and understands you
    C. Always has your back

11. Which of the following statements do you try hardest to live by?
    A. Do unto others, as you would have them do unto you.
    B. Only the educated are free.
    C. You only live once.

12. Which of the following is the most important ingredient in success?
    A. Trying hard
    B. Being organized

C. Getting a good education

13. Do you consider yourself a spiritual/religious person??
    A. No
    B. Yes

14. What do you think is the most important quality in a significant other?
    A. Kindness
    B. Intelligence
    C. Honesty
    D. Sense of humor

15. Which of the following do you like best about your university?
    A. School spirit
    B. Academics
    C. Activities and clubs
    D. Sporting events

16. Which of the following do you think is most important for teachers and students to be able to talk about?
    A. Students' progress on schoolwork
    B. Students' personal problems
    C. Students' future education/career choices

17. If you could have one thing in common with your instructor, which of the following would it be?
    A. Sense of humor
    B. Interest in the same subject matter
    C. Mutual respect
    D. Similar personality

18. Which do you think is more important for students to know about their teachers?
    A. What kind of student they were like when they were in 9th grade
    B. What the expectations are for students in their class

19. Should part of a teacher's role include being a friend to their students?
    A. Yes
    B. No

20. When you aren't discussing school, which of the following do you like to talk to teachers about?
    A. Personal relationships
    B. Politics
    C. Entertainment
    D. Sports

21. I think students learn most when…
    A. The teacher lectures and leads class discussions
    B. Students work independently
    C. Students work in small-groups

Once mentors and mentees filled out the CBoF questionnaire, the research team matched the mentor–mentee pairs based on the CBoF items. For the SII group, matching results including up to five CBoF similarities were sent to the mentors and mentees. An example of highlighted mentor–mentee CBoF similarities in the email reads:

- You both think going to the World Cup would be amazing! Given that you are soccer fans, this might be a fun thing to talk about!
- You both said your ideal trip involves going hiking!
- You are cut from the same cloth - you both described yourselves as hardworking!
- Never give up! You both said trying hard is the most important ingredient in success!
- You both said your university's school spirit is what you like best about your university!

Complete Instruments of Outcome Measures

Intentions to Persist (adopted from Woodcock et al., 2016)

The following questions ask about your *future* career plans. More specifically, we ask you to think about what you will do in your career over the next five years (0 = Definitely Will Not to 10 = Definitely Will). To what extent do you intend to:

1. ...conduct scientific research?
2. ...produce and disseminate scientific scholarship through peer-reviewed academic journal(s)?
3. ...present research findings at scientific conferences?
4. ...Teach scientific content and methods?
5. ...Academically advise and apprentice the next generation of scientific scholars?
6. ...Engage in service to your department, college, and university?
7. ...Advance in your career as an academic/faculty scientist?

Science Self-Efficacy (adopted from Estrada et al., 2011)

This section assesses your confidence in your abilities to function as a scientist in your area. Indicate the extent to which you are confident you can successfully complete the following tasks. Please select the best answer on the scale from 1 (not at all confident) to 5 (absolutely confident).

1. Figure out what data/observations to collect and how to collect them.
2. Create explanations for the results of a scientific study.
3. Develop theories (integrate and coordinate results from multiple studies).

Science Identity (adopted from Estrada et al., 2011)

The following questions ask how you think about yourself and your personal identity. We want to understand how much you think that being a scientist is part of who you are. For the purposes of

this study when you see the word scientist it is intended to mean a professional undertaking research activities in your area of study (e.g. a biologist or a research psychologist). Please select the best answer on the scale from 1 (strongly disagree) to 5 (strongly agree).

1. I have a strong sense of belonging to the community of scientists.
2. I have come to think of myself as a "scientist."
3. I feel like I belong in the field of science.
4. Being a scientist is an important reflection of who I am.

Scientific Community Values (adopted from Estrada et al., 2011)

Please read each description and think about how much each person is or is not like you. Check the answer that best reflects how much the person in the description is like you (1 = Not like me at all to 6 = Very much like me).

1. A person who thinks discussing new theories and ideas between scientists is important.
2. A person who thinks it is valuable to conduct research that builds the world's scientific knowledge.
3. A person who thinks that scientific research can solve many of today's world challenges.
4. A person who feels discovering something new in the sciences is thrilling.

Supplemental Table 1

Instructors' Outcomes as a Function of Treatment Condition (N = 116, n = 58 per group)

	Treatment (SII)	Control
	n (%) or M (SD)	*n (%) or M (SD)*
Persistence Intentions (Pre)	8.29 (1.54)	8.62 (1.21)
Persistence Intentions (Post)	7.88 (2.00)	8.21 (1.59)
Scientific Self-Efficacy (Pre)	4.09 (0.63)	4.12 (0.54)
Scientific Self-Efficacy (Post)	4.09 (0.78)	4.14 (0.71)
Scientific Identity (Pre)	4.21 (0.69)	4.14 (0.60)
Scientific Identity (Post)	4.25 (0.72)	4.21 (0.64)
Scientific Community Values (Pre)	5.51 (0.57)	5.42 (0.56)
Scientific Community Values (Post)	5.52 (0.60)	5.34 (0.69)

Note. SII = social inclusion intervention; The reliability coefficients at pretest (average of T1—T3) and posttest (T4) are as follows: $\alpha_{\text{Persistence Intentions}}$ Pretest = 0.81, Posttest = 0.83; $\alpha_{\text{Scientific Self-Efficacy}}$ Pretest = 0.87, Posttest = 0.90; $\alpha_{\text{Scientific Identity}}$ Pretest = 0.84, Posttest = 0.89; $\alpha_{\text{Scientific Community Values}}$ Pretest = 0.78, Posttest = 0.83. At baseline, the control and SII intervention group did not differ on the pre-intervention variables: $t(114) = 1.28$, $p = .202$ for Persistence Intentions; $t(114) = .28$, $p = .784$ for Scientific Self-Efficacy; $t(114) = .58$, $p = .561$ for Scientific Identity; $t(114) = .86$, $p = .393$ for Scientific Community Value.

Data availability statement:

The data that support the findings of this study are openly available in the Texas Data Repository at https://doi.org/10.18738/T8/DV1GGB
